# A commentary on ‘Electrochemotherapy for advanced cutaneous angiosarcoma: A European register-based cohort study from the International Network for Sharing Practices of electrochemotherapy (InspECT)’

**DOI:** 10.1097/JS9.0000000000001039

**Published:** 2024-01-11

**Authors:** Jinghui Sun, Yanqin Wang, Yuanyuan Li, Juan Li, Min Yan

**Affiliations:** a Department of Dermatology, Shengli Oilfield Central Hospital, Dongying; b Department of Dermatology and Venereology, Dongying People’s Hospital (Dongying Hospital of Shandong Provincial Hospital Croup), Shandong; c Department of Dermatology, Qingpu Branch of Zhongshan Hospital Affiliated to Fudan University, Shanghai, People’s Republic of China

*Dear Editor,*

Angiosarcomas, which account for 1–2% of all head and neck sarcomas, are extremely rare malignancies that penetrate local structures through anaplastic vascular or lymphatic cells^1,2^. Campana *et al*.^3^ conducted a multi-institutional prospective cohort analysis on patients with locally advanced or metastatic cutaneous angiosarcoma (cAS) who received electrochemotherapy (ECT). As a result, ECT should be taken into consideration for advanced cAS since it generates a sustained response rate with little adverse effects. Reading about a prospective cohort spanning multiple institutions and including a sufficient number of patients and follow-ups is a pleasure.

Twenty patients with advanced cAS in the limbs, breast/trunk, or scalp/face were recruited. The median size of the target tumors (*n*=51) was 2.3 cm (range, 1–20). The CTCAE v5.0 and RECIST v1.1 criteria, as well as visual analogue score (VAS) for pain, acceptance of retreatment, and the EQ-5D questionnaire, were recorded after 24 ECT courses were performed utilizing a 1–4 cm treatment safety margin around tumors. The objective response rate was 80%, with a 40% complete response rate. Skin ulceration and discomfort were symptoms of grade-3 poisoning, although there was no significant change in PRO (Patient-Reported Outcome) scores. In all, 13/14 individuals with ulcerated tumors had their bleeding under control. The local progression-free survival (LPFS) was 10.9 months, with a median overall survival of 12.5 months. There were some limitations to this investigation. First, some lingering confounding elements may have gone unnoticed. Older age, tumor size greater than 45 mm, and inability to get clean surgical margins are risk factors associated with a poor prognosis^4^. Second, because ECT was not the only treatment available, there may have been some bias. The study included five patients who had ECT in conjunction with surgical resection and three individuals who received ECT in conjunction with concurrent systemic treatment. Finally, the bias exists since there is no control group.

A more extensive and multicenter study is needed to confirm this finding. Furthermore, to determine the optimal course of action, further in-depth prospective research, clinical trials, and analysis of indications and risk factors in diverse patient subgroups are required. The following aspects can be considered in future research designs: First, do a thorough multicenter analysis to confirm the current findings and investigate indications and risk factors in other patient groupings. Second, a larger number of people with advanced or metastatic cAS are included to ensure that the findings are applicable to all patient populations^5^.

The purpose of this study is to see if ECT, a skin-directed treatment based on cytotoxic chemotherapy mixed with local electric pulses, may be utilized to treat advanced cAS. This issue has far-reaching ramifications for clinical practice. This multi-institutional prospective cohort study provides early evidence that ECT has a high sustained response rate with little side effects and should be evaluated as a treatment option for advanced cAS. Patient tolerance, local hemostasis, and long-term local control are all palliative benefits. Further research is needed to define appropriate scheduling, treatment safety margins, and conjunction with surgery. This proactive thinking provides useful guidance and incentive for the growth of this field of study. In conclusion, ECT has a high sustained response rate with little adverse effects and should be explored for advanced cAS.

## Ethical approval

This manuscript is a comment. Don’t need ethical approval.

## Consent

This manuscript is a comment. Don’t need patients’ consent.

## Source of funding

Not applicable.

## Author contribution

J.S. and Y.W.: study concept or design, data collection, data analysis or interpretation, and writing the paper; Y.L. and J.L.: data collection; M.Y.: study concept or design, writing, and revised the paper.

## Conflicts of interest disclosure

This manuscript is a comment without conflicts of interest.

## Research registration unique identifying number (UIN)

This manuscript is a comment. Don’t need UIN.

## Guarantor

Min Yan.

## Data availability statement

This manuscript is a comment. Don’t need a Data availability statement. However, all the data from the current study are publicly available.

## Provenance and peer review

This manuscript is a comment without being invited.
